# The importance of “Fit”: an interview with Victor Garcia on vascular biology and enjoying the scientific journey

**DOI:** 10.1038/s42003-022-03193-3

**Published:** 2022-03-15

## Abstract

Dr. Victor Garcia is an Assistant Professor at New York Medical College (NYMC) in Valhalla, New York. Dr. Garcia received his PhD from NYMC and completed a post-doctoral research fellowship at Yale University before starting his independent research lab. In this Q&A, Dr. Garcia tells us about his current work in vascular function and disease, the importance of finding a good “Fit” in a project, and highlights parallels between science and art.

**Figure:**
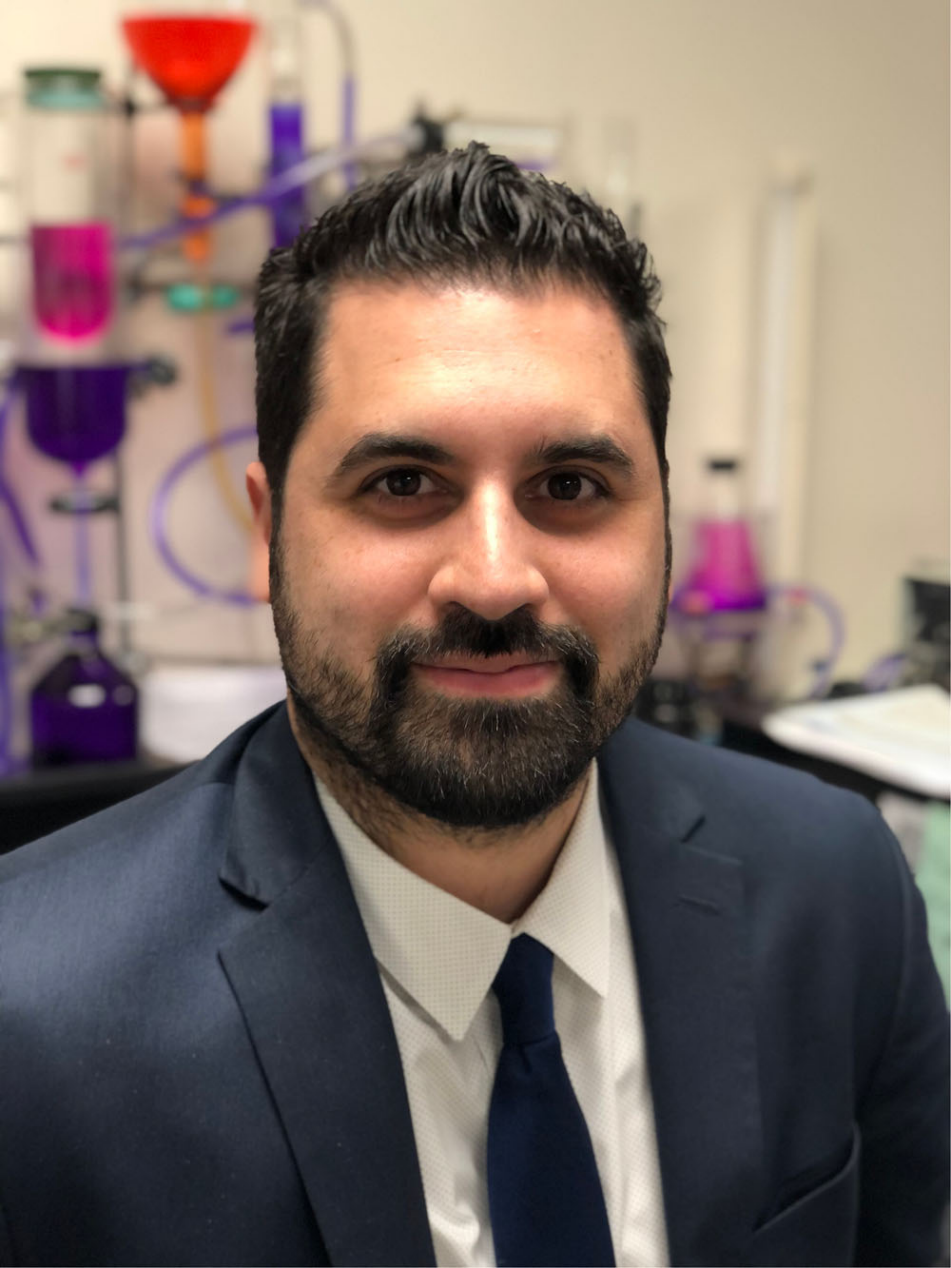
Victor Garcia

**Please tell us about your academic background and research interests**.

My work is focused on the role of various mediators in the regulation of vascular function and disease. These mediators include the vasoactive lipid 20-Hydroxyeicosatetraenoic acid (20-HETE) and the plasminogen activator inhibitor-1 (PAI-1). 20-HETE has long been recognized as a potent lipid of the microcirculation that influences inflammation, blood pressure, and most recently it is implicated in the promotion of insulin resistance, diabetes, and obesity. Recently, our efforts uncovered 20-HETE as a high-affinity ligand for the orphan receptor, GPR75, a Gq-coupled GPCR, expressed across various tissues including the vasculature, endocrine tissues, lung, kidneys, heart, brain, and adipose tissue. With respect to PAI-1, we are interested in its newfound non-proteolytic role as an intracellular endothelial nitric oxide synthase (eNOS) interactor wherein PAI-1 inhibits the synthesis and bioavailability of nitric oxide (NO). Although these two mediators have their own distinct roles, we believe that there is a greater interplay between the 20-HETE/GPR75 and PAI-1/eNOS axes across the vasculature and beyond.

Why is it important to study orphan receptors, like GPR75?

Understanding the role of receptors whether they are considered orphan or deorphanized is incredibly important as they play such a vital role in the activation of molecular pathways. In many cases, these receptors drive and promote the onset and progression of the disease. Moreover, orphan and deorphanized receptors make up a large part of the druggable genome. Therefore, understanding their roles, how they are activated, and how they influence other receptor systems is an incredibly exciting research area. With respect to GPR75 and its ligands (20-HETE and CCL5), there are such a plethora of exciting aspects left to uncover and dissect. For example, our labs (Garcia and Schwartzman at NYMC) in collaboration with Regeneron uncovered that mice deficient in GPR75 and several human GPR75 loss-of-function variants are protected from obesity. Moreover, our studies clearly illustrate that the vasoactive lipid 20-HETE serves as a high-affinity ligand for GPR75. These findings now raise even more questions in my mind like; “How? What molecular mechanism could be at play here? What cells and organ systems are involved? Can we develop and characterize pharmacological receptor blockers targeting GPR75 that protect against obesity?” As we plan and conduct experiments, we are motivated and driven by the fact that targeting this orphan receptor has the potential to alleviate symptoms and diseases associated with GPR75 including hypertension, cancer, diabetes, and obesity. Understanding the fundamental and specific nature of orphan receptors such as GPR75 will hopefully lead to a host of new therapeutics and it is one of the major reasons I get so excited about this field of study.

What do you think are some of the most exciting recent advances in vascular physiology, or what do you hope will be achieved in the next few years?

One of the really fascinating things that we are finally appreciating not just in the field of vascular biology or physiology but across almost every field is how many molecular mechanisms interplay with each other. For example, for decades many research labs were focused on their single molecule of interest, whether it was a ligand, an antagonist, or a protein, and were never able to fully appreciate how that molecule is tied to a greater story and multiple signaling/organ systems. With the array of scientific advances that are now available, we can now open our eyes more and appreciate pathways and systems of 10 or even 20 proteins at once and appreciate the dynamic nature of mechanisms that drive inflammation, hypertension, or obesity, for example. This can get amplified even more when we start to talk about next-generation sequencing, we now go into hundreds of genes and pathways. Not only that, researchers integrate and examine multiple organ systems and try to dissect how they communicate and influence each other under pathological stressors which is also fascinating. We now have more tools than we ever had, from powerful/effective gene silencing, gene overexpression, pharmacological blockers/antagonists, and other hugely sophisticated assays which have kicked up the pace on how we conduct science. With that said, I honestly feel that oftentimes we go too fast and miss out on being able to appreciate unique clues and the nuances of our findings/data. Taking your time and running away from the pressure to publish something immediately can really get you to better appreciate and gain deeper insights into what is in front of you and in many cases, these clues can drive the future of your research projects or program into exciting new avenues. To any young or even veteran scientist, “Take your time! Enjoy and take in the process slowly. Let your imagination run wild and most of all, make mistakes.” Learn from those mistakes and grow as a person and scientist.

How has your experience as a first-generation college student influenced your own approach to mentoring and outreach?

Now that I look back at it, being a first-generation college student has had such a huge impact on the way I see and approach so many things. Being financially disadvantaged and from an underrepresented minority throughout my entire academic career, has really made me value and take advantage of every and any opportunity that came along. The Wight Foundation, of Newark, NJ was really the first opportunity that changed my life in such a powerful way. The foundation provides scholarships so that underrepresented minorities can attend boarding schools for their high school education. They do a phenomenal job of not only preparing students for this huge step but really honing in on whether or not the prospective boarding school fits best for the student. This concept of “Fit” is something I talk about constantly as I mentor and conduct scientific outreach simply because the moment you “Fit”, whether it means you love a project, your work environment, your colleagues, or your mentor; that is the moment when you really thrive and can have incredible success in your career. Many of us try to force our way into what other people think is best for us or into situations where we are constantly uncomfortable. Now while breaking through discomfort can be powerful and in many instances facilitates personal growth, I tell every student I meet that if you hate coming into the lab or it just doesn’t feel good to you to be working where you are, please find something or somewhere else where you can thrive. I have been incredibly fortunate to have some incredible mentors throughout my academic career including Dr. Windfelder (Drew University: undergrad), Dr. Schwartzman (NYMC: PhD training), and Dr. Sessa (Yale University: post-doctoral training). I cannot thank them enough for their time, their support, and their continued mentorship that has shaped me as a scientist and developed my career. Ultimately, my current goal is to provide as many research and mentoring opportunities as I can to students from all backgrounds and levels of experience, emphasizing the importance of “Fit” and making sure they land in a place where they love what they work on and are proud of what they do.

You’re also an active stencil artist (PaperMonster). Do you see any parallels between your artistic and scientific interests?

Art and science have incredible parallels at almost every single level. For example, first and foremost you have to know your history. By this I mean that you have to have an idea of the people that came before you and what they created, painted, or designed. The same goes for science. The foundational pieces of knowledge that have been published across the world set the stage for new discoveries and provide that spark for inspiration and curiosity as we plan, conduct, and analyze experiments. Once you have a strong grasp of that history, you not only feel more comfortable with the tools at your disposal to create, you can begin to take greater steps forward to blaze your own trail and create a name for yourself in your respective field. No piece of artwork is ever fully completed or perfect and the same goes for a manuscript. The life of an artist and scientist is all about training, growth, and in many cases rejection. At times, the process is greater and more important than the final product. Lastly, in both art and science, you leave behind a legacy defined by your name and work whether they are beautiful works of art or incredible scientific discoveries that can have an everlasting impact.

*This interview was conducted by Associate Editor George Inglis*.

