# Publisher Correction: The importance of “Fit”: an interview with Victor Garcia on vascular biology and enjoying the scientific journey

**DOI:** 10.1038/s42003-022-03386-w

**Published:** 2022-05-05

Correction to: *Communications Biology* 10.1038/s42003-022-03193-3, published online 15 March 2022.

In this Q&A the affiliation details for Victor Garcia were incorrectly given as “Associate Professor at New York Medical College” but should have been “Assistant Professor at New York Medical College”.

Both errors have now been corrected in the HTML and PDF versions of the Q&A.

